# Optimising prescribing in older adults with multimorbidity and polypharmacy in primary care (OPTICA): cluster randomised clinical trial

**DOI:** 10.1136/bmj-2022-074054

**Published:** 2023-05-24

**Authors:** Katharina Tabea Jungo, Anna-Katharina Ansorg, Carmen Floriani, Zsofia Rozsnyai, Nathalie Schwab, Rahel Meier, Fabio Valeri, Odile Stalder, Andreas Limacher, Claudio Schneider, Michael Bagattini, Sven Trelle, Marco Spruit, Matthias Schwenkglenks, Nicolas Rodondi, Sven Streit

**Affiliations:** 1 Institute of Primary Health Care (BIHAM), University of Bern, Bern, Switzerland; 2 Department of General Internal Medicine, Inselspital, Bern University Hospital, University of Bern, Bern, Switzerland; 3 Institute of Primary Care, University of Zurich and University Hospital Zurich, Zurich, Switzerland; 4 CTU Bern, University of Bern, Bern, Switzerland; 5 mfe Haus and Kinderärzte Schweiz, Bern, Switzerland; 6 Department of Information and Computing Sciences, Utrecht University, Utrecht, Netherlands; 7 Public Health and Primary Care (PHEG), Leiden University Medical Center, Leiden University, Leiden, Netherlands; 8 Leiden Institute of Advanced Computer Science (LIACS), Faculty of Science, Leiden University, Leiden, Netherlands; 9 Institute of Pharmaceutical Medicine (ECPM), University of Basel, Basel, Switzerland; 10 Epidemiology, Biostatistics and Prevention Institute (EBPI), University of Zurich, Zurich, Switzerland

## Abstract

**Objective:**

To study the effects of a primary care medication review intervention centred around an electronic clinical decision support system (eCDSS) on appropriateness of medication and the number of prescribing omissions in older adults with multimorbidity and polypharmacy compared with a discussion about medication in line with usual care.

**Design:**

Cluster randomised clinical trial.

**Setting:**

Swiss primary care, between December 2018 and February 2021.

**Participants:**

Eligible patients were ≥65 years of age with three or more chronic conditions and five or more long term medications.

**Intervention:**

The intervention to optimise pharmacotherapy centred around an eCDSS was conducted by general practitioners, followed by shared decision making between general practitioners and patients, and was compared with a discussion about medication in line with usual care between patients and general practitioners.

**Main outcome measures:**

Primary outcomes were improvement in the Medication Appropriateness Index (MAI) and the Assessment of Underutilisation (AOU) at 12 months. Secondary outcomes included number of medications, falls, fractures, and quality of life.

**Results:**

In 43 general practitioner clusters, 323 patients were recruited (median age 77 (interquartile range 73-83) years; 45% (n=146) women). Twenty one general practitioners with 160 patients were assigned to the intervention group and 22 general practitioners with 163 patients to the control group. On average, one recommendation to stop or start a medication was reported to be implemented per patient. At 12 months, the results of the intention-to-treat analysis of the improvement in appropriateness of medication (odds ratio 1.05, 95% confidence interval 0.59 to 1.87) and the number of prescribing omissions (0.90, 0.41 to 1.96) were inconclusive. The same was the case for the per protocol analysis. No clear evidence was found for a difference in safety outcomes at the 12 month follow-up, but fewer safety events were reported in the intervention group than in the control group at six and 12 months.

**Conclusions:**

In this randomised trial of general practitioners and older adults, the results were inconclusive as to whether the medication review intervention centred around the use of an eCDSS led to an improvement in appropriateness of medication or a reduction in prescribing omissions at 12 months compared with a discussion about medication in line with usual care. Nevertheless, the intervention could be safely delivered without causing any harm to patients.

**Trial registration:**

NCT03724539Clinicaltrials.gov NCT03724539

## Introduction

Inappropriate polypharmacy in older adults is a major driver of healthcare related harm.^1^ ^2^ It is associated with negative health outcomes, such as adverse drug events, falls, and functional decline in activities of daily living.^3^ ^4^ ^5^ ^6^ Patients with multiple chronic conditions (multimorbidity^7^) and polypharmacy, defined as the use of five or more drugs,^8^ are at an increased risk of inappropriate polypharmacy, such as inappropriate prescribing and prescribing omissions.^9^ ^10^ ^11^ ^12^ This highlights the need for reducing inappropriate polypharmacy, and medication reviews represent one approach to this. Primary care settings, characterised by long term patient-provider relationships, lend themselves as ideal settings for medication reviews. Conducting medication reviews is, however, complex and time consuming, and the evidence for medication review interventions is mixed.^13^ ^14^ ^15^

Medication review interventions, such as those based on the Screening Tool of Older Persons potentially inappropriate Prescription (STOPP) and Screening Tool to Alert doctors to the Right Treatment (START) criteria—evidence based criteria to inform prescribing in older adults^16^ ^17^—can support general practitioners to optimise prescribing. These criteria have been shown to be effective in improving quality of prescribing and some patient outcomes.^18^ ^19^ In the context of digitalisation, use of electronic clinical decision support systems (eCDSS) based on the STOPP/START criteria, such as the Systematic Tool to Reduce Inappropriate Prescribing Assistant (STRIPA), is a promising way forward.^20^ ^21^ ^22^ In recent years, different eCDSS for optimising drug use have been tested.^23^ ^24^ ^25^ Systems based on the STOPP/START criteria were used in two randomised clinical trials conducted in the inpatient setting.^19^ ^26^ However, evidence from primary care settings is lacking.

The Optimising PharmacoTherapy In the multimorbid elderly in primary CAre (OPTICA) trial tested the hypothesis that, in older adults with multimorbidity and polypharmacy, the use of an eCDSS for optimising drug therapy by general practitioners improves appropriateness of medication and reduces prescribing omissions compared with standard care.

## Methods

### Trial design

The protocol of the OPTICA trial was published previously.^27^ We conducted a cluster randomised controlled trial with 43 general practitioners as clusters.^28^ The trial was conducted from 2018 to 2021.

### Recruitment and participants

General practitioners had to be participating in the Family medicine ICPC Research using Electronic medical records (FIRE) project throughout the trial.^29^ This allowed electronic data exports from their practices to the trial database and the eCDSS.

General practitioners recruited eight to 10 eligible patients (supplementary figure A) by using random screening lists generated from their practice’s electronic health record data.^30^ If needed, more than one screening list with 20 patients each was provided to general practitioners. General practitioners obtained written informed consent from all participants or their legal representatives before enrolment in the study. For patients with cognitive impairment, written informed consent was obtained from their legal representative.

Patients were aged ≥65 years, were taking five or more long term medications (≥90 days) and had at least three chronic conditions on the basis of on ICPC-2 (international classification of primary care, 2nd edition) coding or general practitioners’ clinical judgement. To maximise the generalisability of the study population, we kept exclusion criteria to a minimum. Exclusion criteria were inability to provide consent and participation in a different intervention study.

### Randomisation

Clusters were randomised after enrolment of patients for each cluster was completed. Participating general practitioners were randomised centrally in a web based system (REDCap).^31^ ^32^ We used a one to one ratio with unstratified block randomisation and randomly varying block sizes of two and four.

### Trial procedures

#### Intervention group

As previously reported,^27^ general practitioners used the intervention at the individual patient level. The intervention consisted of a structured six step medication review using STRIPA, a web based electronic clinical decision support system based on the STOPP/START criteria version 2 (appendix 1).^17^ ^33^ For the purpose of the OPTICA trial, STRIPA was adapted to the primary care setting (for example, use of ICPC-2 codes instead of ICD (international classification of diseases) codes for the coding of diagnoses). In addition to detecting potential overuse, underuse, and misuse of drugs, STRIPA generated recommendations to prevent drug-drug interactions and inappropriate dosages. The one time intervention consisted of six steps. (1) Data on medications, chronic conditions, laboratory values, and vital data were imported to STRIPA. (2) General practitioners verified and adapted the recorded information. (3) General practitioners used the drag/drop function to link medications and conditions. (4) General practitioners ran the medication review. (5) General practitioners decided which recommendations to move forward with. (6) At the next appointment, general practitioners implemented shared decision making with patients. General practitioners in the intervention group received a training video and written material on how to use STRIPA and conduct the shared decision making.

#### Control group

Patients in the control group had a discussion about medication with their general practitioner in line with usual care. General practitioners were asked not to deviate from their usual practice.

### Blinding

General practitioners were blinded during the screening and recruitment of patients to limit biased selection of patients. General practitioners in the control group remained partially blinded, as they did not know the intervention procedure. Patients in the control group remained blinded owing to the discussion with their general practitioner. The data collectors and study assessors were fully blinded. Blinding of the trial statistician was not feasible because the data export contained information on the study groups.

### Outcomes

#### Primary outcome measures

Appropriateness of medication was the primary outcome. To account for the multi-dimensionality of this construct and to capture both over-prescribing and under-prescribing, we used two primary outcome measures: the Medication Appropriateness Index (MAI) and the Assessment of Underutilisation (AOU) (both at 12 months). The MAI allows assessment of the appropriateness of prescriptions, and the AOU measures the number of prescribing omissions.^34^ ^35^ ^36^ We assessed the AOU for each non-acute condition of the patients and the MAI for each long term medication. We used the 10 item version of the MAI. However, we excluded the MAI’s cost effectiveness item for feasibility reasons (appendix 2). This resulted in a score from 0 to 17 for each medication (with a higher score representing greater inappropriateness). We defined improvement in the MAI conservatively as a decrease of ≥1 points for all medications used by the patient, which represents an increase in appropriateness of medication. For non-acute diagnoses, the AOU assessed no prescribing omission, a marginal omission (for example, use of non-drug treatment), or an omission of an indicated medication.^37^ ^38^ ^39^ Improvement in the AOU was a reduction of ≥1 prescribing omissions considering all chronic conditions of the patient. The inter-rater reliability assessments showed moderate agreement regarding the MAI assessments (agreement of ratings=69%, Kendall’s coefficient of concordance W^40^=0.61, Cohen’s κ=0.54) and substantial agreement regarding the AOU assessments (agreement of ratings=94%, Kendall’s coefficient of concordance W=0.77), which validated the use of both assessments.^40^

#### Secondary outcomes

Main secondary outcomes were patients’ long term medications (degree of polypharmacy), appropriateness of medication (measured by the ordinal MAI), the number of prescribing omissions, the number of falls and fractures, and quality of life as measured by the telephone version of the EQ-5D-5L questionnaire.^41^ ^42^ The percentage of prescribing recommendations accepted was a secondary outcome reported to describe fidelity of implementation. Next, we assessed patients’ willingness to have medications deprescribed at baseline and reported it in this manuscript. The secondary outcomes for the health economic analyses conducted alongside the trial and reported separately were health services use (formal care received—for example, number of general practitioner and specialist visits), informal care received (for example, unpaid care work by relatives/friends), survival, quality adjusted life years,^43^ and direct medical costs accrued in one year.

### Data collection

We collected data at baseline, six months, and 12 months. The data on medications, diagnoses, laboratory values, and vital signs were imported from the electronic health records of patients through the FIRE database.^29^ Owing to variation in data exports from different electronic health records software programs used by general practitioners (reporting of medications and diagnoses at every encounter versus reporting only when a change was made in the record), fewer medications and diagnoses were recorded for some patients for the observed study period. However, this does not mean that patients did not fulfil the inclusion and exclusion criteria, as general practitioners had screened and verified those as part of the recruitment process. Owing to the substantial amount of missing data in the variables needed to assess the primary outcomes (~35%), the study team collected missing information from participating general practitioners. After this additional data collection, 13% and 18% of patients had missing information on MAI and AOU improvement, respectively, between baseline and follow-up 2 (supplementary tables A and B). We collected data on quality of life, health services use, and falls and fractures through phone calls with patients or legal representatives. General practitioners reported safety information on adverse events including death. We asked general practitioners in the intervention group to report information on the implementation (or not) of prescribing recommendations. All data were coded and kept confidential.

### Sample size calculation

We calculated the sample size needed to test for superiority of the two primary outcome measures and used the Bonferroni approach to account for multiple testing. We assumed that 35% and 60% of patients would have an improvement in the MAI and that 10% and 30% would have an improvement in the AOU in the control and intervention group, respectively. On the basis of a two sample comparison of proportions, a pre-specified number of general practitioner clusters of 40 (20 per arm), and a conservative intracluster correlation coefficient of 0.05 (values of 0.01-0.05 are typically found for binary outcomes in older people^44^), we needed seven patients per cluster to detect a difference in the proportion of improvement in the MAI score of 25% between the two groups with a power of 90% at a two sided α level of 0.025. Using the same assumption for the AOU, we also needed seven patients per cluster to detect a difference of 20%. This results in a total sample size of 280 patients (140 per arm). This sample size provides 81% power to detect a significant improvement in both the MAI score and the AOU index. To account for attrition due to dropout or death (15% estimated), we enlarged the number of patients per cluster to eight to 10, with a final sample size of 320 patients total (160 per group).

### Statistical analysis

We described the sociodemographic characteristics of general practitioners. We described the characteristics of patients and presented them by group. We analysed the number of prescribing recommendations generated and implemented descriptively. In all the model based analyses, we used multiple imputed data. We used multiple imputation by chained equations to impute missing values in co-primary outcomes (MAI score and prescribing omissions measured using the AOU index) at baseline, six months, and 12 months. Imputation models were based on all baseline characteristics of the patients (age, gender, education level, smoking status, alcohol consumption, permanent nursing home stay, number of falls, number of hospital admissions, number of chronic medications, number of chronic conditions, quality of life predicted utilities at baseline, EQ-5D visual analogue scale, and patients’ willingness to have medications deprescribed) and the general practitioners’ characteristics (randomisation group, practice form, practice size, canton of the practice, general practitioners’ work experience in years, and practice location). We did not account for within cluster correlation in the imputation model. We used these imputed variables to impute all the secondary outcomes at six and 12 months. We used predictive mean matching (pmm) and logit models to impute non-binary and binary variables, respectively. We generated 50 imputed datasets, which we analysed using Rubin’s rules to combine results across datasets.^45^

We tested the primary outcomes separately, declaring success if at least one was statistically significant at the Bonferroni corrected, two sided α level of 0.025. We used generalised estimating equation models with robust standard errors to account for clustered data, which yields population averaged effects.^46^ ^47^ ^48^ For binary outcomes, we calculated odds ratios by using a binomial distribution and a logit link. For count outcomes, we calculated incidence rate ratios by using a negative binomial distribution and a log link. For continuous outcomes, we calculated mean differences by using a Gaussian distribution and an identity link. We did several pre-specified subgroup comparisons at general practitioner and patient level through the models described above (appendix 3), with additional interaction terms between randomisation group and binary subgroup indicators. Odds ratios, incidence rate ratios, or mean differences for randomised comparisons are shown for each subgroup together with a P value for interaction. For the comparison of safety outcomes, we used a robust generalised estimating equation model with a binomial distribution and a logit link.

In secondary analyses, we re-ran all models with the per protocol set of patients. Here, we excluded all patients for whom fewer than three chronic conditions and fewer than five long term medications had been recorded during the study period, as well as all clusters that included fewer than four participants. In addition, we did a post hoc relaxed per protocol analysis in which only patients with no chronic condition and no long term medication recorded and clusters with fewer than four patients were excluded. In an additional sensitivity analysis, we adjusted models for potential confounders because cluster randomisation may lead to imbalances in baseline characteristics between groups. Finally, we did an aggregated data analysis at the general practitioner level.

Owing to overdispersion of the secondary count outcomes, a deviation from the protocol had to be made and was prespecified in the statistical analysis plan. We used a robust generalised estimating equation model with a negative binomial distribution and a log link rather than the too restrictive random effects Poisson model. Furthermore, the generalised estimating equation approach is more consistent with the other analyses than is the random effects model. We considered continuous secondary outcomes (MAI score, AOU index) as count data and analysed them using a generalised estimating equation model with a negative binomial distribution and a log link owing to their skewed distribution. As the MAI score consists only of integers with a high proportion of zeros, its distribution cannot be considered as gaussian. Further minor deviations from the statistical analysis plan are reported in appendix 4. We used Stata version 17.0 for all analyses. We report the results in line with the Consolidated Standards of Reporting Trials extension for cluster trials.^49^

### Patient and public involvement

No patients were involved in setting the research question or the outcome measures. General practitioners and patients aged ≥65 years with multimorbidity and polypharmacy were represented in the Safety and Data Monitoring Board. General practitioners and patients who participated in the trial received newsletters throughout the trial.

## Results

**Fig 1:**
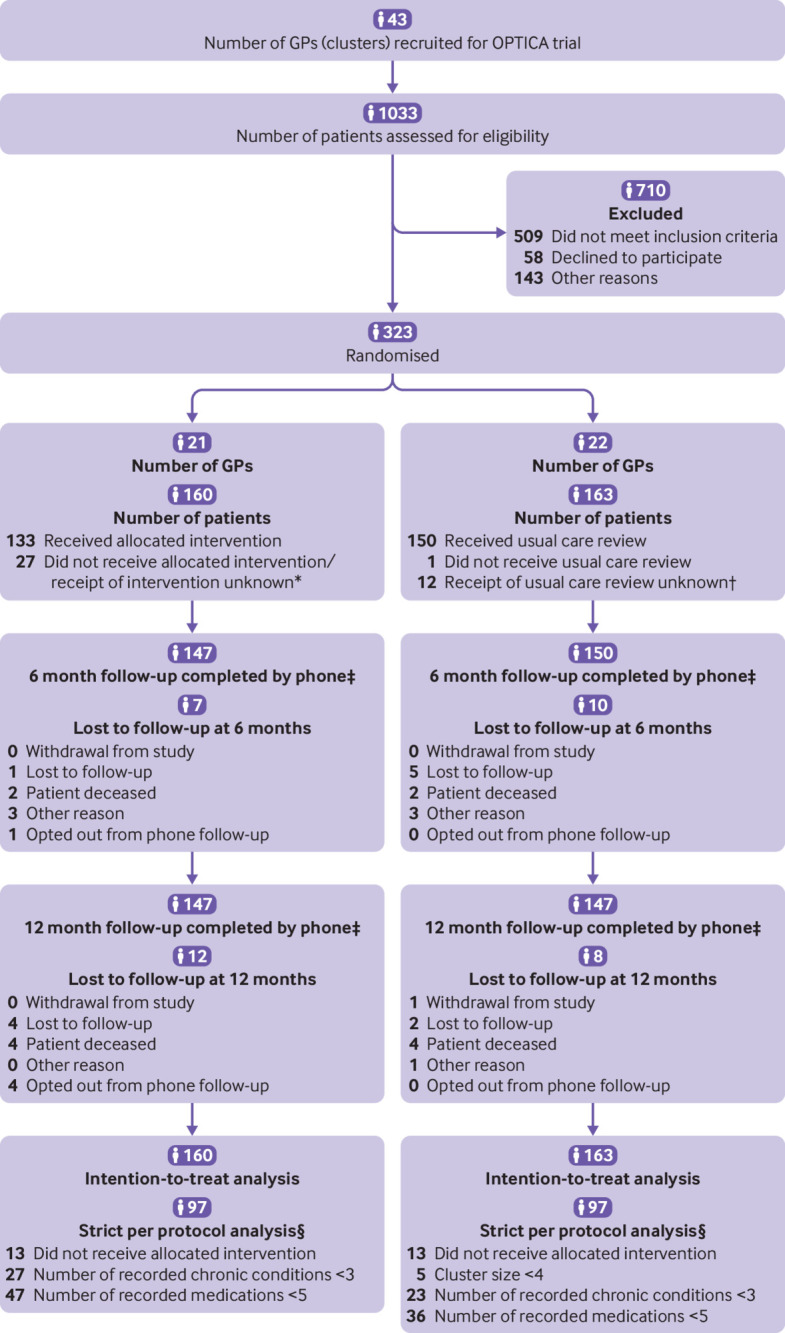
CONSORT patient flowchart. *For these patients, drag/drop function in Systematic Tool to Reduce Inappropriate Prescribing Assistant (STRIPA) had not been used or was reset after intervention. †Reasons are that patients did not see their general practitioner (GP) or had other urgent healthcare needs that had to be prioritised. ‡Referring to follow-up calls by phone. Time windows for these phone calls were +15 days at baseline, +/–30 days at six month follow-up, and +/–30 days at 12 month follow-up. For all patients, except those who withdrew from study, available data from FIRE database could be used (provided that patient continued seeing same GP). §Multiple criteria can apply. Multiple imputed data were used for analyses

Forty three general practitioners working in Swiss primary care practices participated in the OPTICA trial (supplementary figure A).^30^ Between January 2019 and February 2020, 323 older adults with multimorbidity and polypharmacy (median age 77 (interquartile range 73-83) years; 45% women) gave their informed consent. Twenty one general practitioners with 160 patients were randomised to the intervention group and 22 general practitioners with 163 patients to the control group (fig 1). During the 12 month follow-up period, 12 (4%) patients died, 12 (4%) patients were lost to follow-up and could not be reached by phone for the data collection, five (2%) patients opted out of the data collection by phone but agreed to be followed-up via the FIRE database, and one patient withdrew from the study.

Participating general practitioners had a median age of 53 (44-58) years and median work experience of 14 (7-21) years, and 21% were female. At baseline, patients had a median number of long term medications of 7 (4-10) and a median number of chronic diagnoses of 7 (4-10). The baseline characteristics of patients were similar in the two groups, except for appropriateness of medication, which was greater in the control group (as shown by the lower total MAI) (table 1).

**Table 1:** Characteristics of general practitioners (GPs) and patients at baseline. Values are numbers (percentages) unless stated otherwise

	All clusters	Control group	Intervention group
**General practitioners (clusters)**	**(n=43)**	**(n=22)**	**(n=21)**
No of patients per cluster:			
<4	2 (5)	2 (9)	0 (0)
4-7	9 (21)	3 (14)	6 (29)
≥8	32 (74)	17 (77)	15 (71)
Practice location:			
Rural	21 (49)	12 (55)	9 (43)
Urban/suburban	22 (51)	10 (45)	12 (57)
Median (IQR) age, years	53 (44-58)	54 (44-55)	51 (44-58)
Gender:			
Male	34 (79)	16 (73)	18 (86)
Female	9 (21)	6 (27)	3 (14)
Median (IQR) work experience as GP, years	14 (7-21)	16 (7-21)	12 (8-22)
Median (IQR) No of consultations per workday	25 (20-28)	25 (22-30)	25 (20-25)
Practice form:			
Individual practice	7 (16)	5 (23)	2 (10)
Group practice	36 (84)	17 (77)	19 (90)
Median (IQR) No of GPs in group practices	3 (2-4)	3 (2-4)	4 (3-5)
**Patients**	** (n=323)**	** (n=163)**	** (n=160)**
Age categories:			
65-74 years	111 (34)	58 (36)	53 (33)
75-84	150 (46)	75 (46)	75 (47)
≥85	62 (19)	30 (18)	32 (20)
Median (IQR) age, years	77 (73-83)	77 (73-83)	77 (73-83)
Gender:			
Male	177 (55)	88 (54)	89 (56)
Female	146 (45)	75 (46)	71 (44)
Highest education level:			
Less than mandatory schooling	4 (1)	1 (1)	3 (2)
Mandatory schooling	118 (37)	62 (38)	56 (35)
High school degree or apprenticeship	145 (45)	70 (43)	75 (47)
University or equivalent	45 (14)	23 (14)	22 (14)
Other	2 (1)	0 (0)	2 (1)
Smoking status:			
Smoker	33 (10)	14 (9)	19 (12)
Past smoker	140 (43)	65 (40)	75 (47)
Non-smoker	142 (44)	77 (47)	65 (41)
Alcohol consumption in 6 months before study enrolment	196 (61)	97 (60)	99 (62)
Median (IQR) alcohol consumption in 6 months before study enrolment, units per week	2 (1-7)	2 (1-7)	2 (1-7)
Permanent stay in nursing home	24 (7)	9 (6)	15 (9)
Cognitive impairment	12 (4)	7 (4)	5 (3)
Falls in 6 months before study enrolment	58 (18)	27 (17)	31 (19)
Median (IQR) No of falls in 6 months before study enrolment	1 (1-1)	1 (1-1)	1 (1-1)
Median (IQR) No of hospital admissions in 6 months before study enrolment	0 (0-0)	0 (0-0)	0 (0-0)
Median (IQR) No of long term medications at baseline	7 (4-10)	8 (5-10)	7 (4-10)
Median (IQR) No of chronic conditions at baseline	7 (4-10)	7 (4-10)	6 (4-9)
Median (IQR) quality of life*	75 (60-80)	70 (60-80)	75 (60-80)
Median (IQR) EQ-5D-5L utilities at baseline	0.89 (0.80-0.96)	0.89 (0.81-0.94)	0.89 (0.77-0.97)
Unwilling to have medications deprescribed†	36 (11)	19 (12)	17 (11)
Median (IQR) total MAI‡ score at baseline	12 (2-38)	5 (0-38)	15 (4-38)
Median (IQR) averaged MAI score at baseline§	1.9 (0.2-5.2)	0.6 (0.0-4.9)	3.0 (0.5-5.4)
Median (IQR) total No of prescribing omissions¶	1 (0-2)	1 (0-2)	1 (0-1)
Median (IQR) averaged No of prescribing omissions**	0.1 (0.0-0.2)	0.1 (0.0-0.2)	0.1 (0.0-0.2)

IQR=interquartile range; MAI=Medication Appropriateness Index.

Missing values: 0 for all GP characteristics, except for number of consultations per day (2%); 0 for all patient characteristics except for education level (3%), smoking status (2%), smoking consumption (2%), living situation (2%), falls (4%), number of hospital admissions (3%), quality of life (8%), both primary outcomes MAI and Assessment of Underutilisation (AOU) at baseline (7%).

* Measured by visual analogue scale of European Quality of Life-5 Dimensions questionnaire (EQ-VAS). Values range from 0 to 100, with higher values indicating higher quality of life.

† Measured by agreement with statement “I would be willing to stop one or more of my medicines if my doctor said it was possible” of revised Patients’ Attitudes Towards Deprescribing (rPATD).^50^ Dichotomized by agree/strongly agree (yes) versus don’t know/disagree/strongly disagree (no). Results are presented only for patients for whom patient version of rPATD was used.

‡ Adapted from Samsa, et al.^36^ Sum of all MAI scores of each medication. Each individual MAI score ranges from 0 to 17, with higher values indicating greater inappropriateness.

§ Averaged by number of chronic medications.

¶ Measured by AOU based on Jeffery et al.^38^ Count of prescribing omissions for chronic conditions at baseline.

** Averaged by the number of chronic conditions.

An average of 5.4 prescribing recommendations per patient were generated, of which an average of 3.7 were STOPP or START recommendations (table 2). On average, 1.0 START or STOPP recommendations were implemented per patient in the intervention group, with 58.5% of patients having had at least one recommendation implemented. Table 3 shows the most common prescribing recommendations made by STRIPA. General practitioners most commonly reported the following reasons why prescribing recommendations were not implemented: belief that the current prescriptions were beneficial, the recommendation was not suitable for patients, and bad experiences with previous medication changes (supplementary table C).

**Table 2:** Recommendations generated by Systematic Tool to Reduce Inappropriate Prescribing Assistant (STRIPA) to optimise prescribing in older adults (n=133)*

	Estimate	Range
**Generated prescribing recommendations (n=704)**		
No (%) patients with ≥1 prescribing recommendation†	130/133 (98)	-
Mean (SD) No of prescribing recommendations per patient†	5.4 (3.2)	1-21
Mean (SD) No of START or STOPP recommendations per patient‡	3.7 (1.8)	0-11
STOPP recommendations	2.3 (1.3)	0-7
START recommendations	1.3 (1.2)	0-6
**Implementation of prescribing recommendations** §		
At patient level:		
No (%) patients with ≥1 prescribing recommendation reported to have been implemented†	31/53 (58)	-
Mean (SD) No of recommendations reported to have been implemented per patient†	1.0 (1.2)	-
At recommendation level:		
No (%) STOPP recommendations implemented	31/112 (28)	-
No (%) START recommendations implemented	11/77 (14)	-

SD=standard deviation; START=Screening Tool to Alert to Right Treatment; STOPP=Screening Tool of Older Persons’ Prescriptions.

* Information collected from STRIPA for 133/160 patients in intervention group for whom drag/drop function in STRIPA had been used as part of intervention. All patients for whom information could be retrieved from STRIPA had ≥1 recommendation to start or stop one or several of their medications.

† Includes recommendations to stop and start medications, adapt dosage of potentially inappropriate prescriptions, or flag drug-drug interactions.

‡ All patients for whom information could be retrieved from STRIPA had ≥1 recommendation to start or stop one or several of their medications.

§ This information was reported by 7 general practitioners from OPTICA intervention group about 53 patients, which explains lower denominator.

**Table 3:** Most common prescribing recommendations generated by Systematic Tool to Reduce Inappropriate Prescribing Assistant of 704 recommendations made

Recommendation type	Description^16^	Frequency (%)
START I1	Seasonal trivalent influenza vaccine annually	121 (17.2)
STOPP B6	Loop diuretic as first line treatment for hypertension (safer, more effective alternatives available)	38 (5.4)
START A2	Aspirin (75-160 mg once daily) in presence of chronic atrial fibrillation, in which vitamin K antagonists or direct thrombin inhibitors or factor Xa inhibitors are contraindicated	33 (4.7)
START E3	Vitamin D supplement in patients with known osteoporosis and previous fragility fracture(s) and/or bone mineral density T scores more than −2.0 at multiple sites	21 (3.0)
START E4	Bone antiresorptive or anabolic therapy (eg, bisphosphonate, strontium ranelate, teriparatide, denosumab) in patients with documented osteoporosis, in whom no pharmacological or clinical status contraindication exists (bone mineral density T scores >2.5 at multiple sites) and/or previous history of fragility fracture(s)	18 (2.6)
START A3	Antiplatelet therapy (aspirin or clopidogrel or prasugrel or ticagrelor) with documented history of coronary, cerebral, or peripheral vascular disease	17 (2.4)
START B1	Regular inhaled β2 agonist or antimuscarinic bronchodilator (eg, ipratropium, tiotropium) for mild to moderate asthma or COPD	15 (2.1)
START A7	β blocker with ischaemic heart disease.	14 (2.0)
STOPP C3	Aspirin, clopidogrel, dipyridamole, vitamin K antagonists, direct thrombin inhibitors, or factor Xa inhibitors with concurrent significant bleeding risk (ie, uncontrolled severe hypertension, bleeding diathesis, recent non-trivial spontaneous bleeding) (high risk of bleeding)	14 (2.0)
START A6	ACE inhibitor with systolic heart failure and/or documented coronary artery disease	12 (1.7)
STOPP B11	ACE inhibitors or ARBs in patients with hyperkalaemia	9 (1.3)
STOPP B9	Aldosterone antagonists (eg, spironolactone, eplerenone) with concurrent potassium conserving drugs (eg, ACE inhibitors, ARBs, amiloride, triamterene) without monitoring of serum potassium (risk of dangerous hyperkalaemia ( >6.0 mmol/L); serum potassium should be monitored regularly (ie, at least every 6 months))	8 (1.1)
START H2	Laxatives in patients receiving opioids regularly	8 (1.1)
STOPP H2	Non-steroidal anti-inflammatory drug in patients with established hypertension (risk of exacerbation of hypertension) or heart failure (risk of exacerbation of heart failure)	7 (1.0)
START E2	Bisphosphonates and vitamin D and calcium in patients taking long term systemic corticosteroid therapy	7 (1.0)
START B3	Home continuous oxygen with documented chronic hypoxaemia (ie, pO_2_<8.0 kPa or 60 mm Hg or SaO_2_<89).	6 (0.9)
STOPP C6	Antiplatelet agents with vitamin K antagonist, direct thrombin inhibitor, or factor Xa inhibitors in patients with stable coronary, cerebrovascular, or peripheral arterial disease without clear indication for anticoagulant therapy (no added benefit from dual therapy)	5 (0.7)
STOPP B4	β blocker with symptomatic bradycardia (<50/min), type II heart block, or complete heart block (risk of profound hypotension, asystole)	5 (0.7)
STOPP F3	Drugs likely to cause constipation (eg, antimuscarinic/anticholinergic drugs, oral iron, opioids, verapamil, aluminium antacids) in patients with chronic constipation in whom non-constipating alternatives are appropriate (risk of exacerbation of constipation)	5 (0.7)

ACE=angiotensin converting enzyme; ARB=angiotensin receptor blocker; COPD=chronic obstructive pulmonary disease.

Recommendations that were generated ≥5 times during study are shown.

### Primary outcome measures

In all, 82% (n=265) of patients had information on the MAI both at baseline and at the 12 month follow-up, and 87% (282) had information on prescribing omissions as assessed using the AOU for both time points (supplementary table A). Thirty seven per cent (119) of patients had an improvement in the MAI between baseline and the 12 month follow-up, and 12% (38) had an improvement in the number of prescribing omissions (supplementary table B). The development of the MAI score and the number of prescribing omissions are shown by time point in supplementary figures B-D and supplementary table D.

The analyses compared the intervention and control groups. In the intention-to-treat analysis, the odds ratio for an improvement in the MAI score (decrease by ≥1 point) between baseline and the 12 month follow-up was 1.05 (95% confidence interval 0.59 to 1.87) and the odds ratio for an improvement in the AOU (≥1 prescribing omission less) was 0.90 (0.41 to 1.96) (table 4). The sensitivity analysis adjusting for baseline MAI and AOU values (supplementary table E), the sensitivity analysis adjusting for subgroup variables (supplementary table E), the subgroup analyses (supplementary figure E), the aggregated data analysis (supplementary table F), and the analysis based on the available case data (supplementary table G) also showed inconclusive results.

**Table 4:** Comparison of outcomes between intervention and control groups

	Control (n=163)	Intervention (n=160)	Effect size (95% CI)	P value
**Primary outcomes**				
No (%) with improvement in MAI score between baseline and 12 month follow-up	67 (41); multiple imputation used for 20/163 patients	68 (43); multiple imputation used for 21/160 patients	OR 1.05 (0.59 to 1.87)	0.87
No (%) with improvement in number of prescribing omissions between baseline and 12 month follow-up	28 (17); multiple imputation used for 30/163 patients	24 (15); multiple imputation used for 28/160 patients	OR 0.90 (0.41 to 1.96)	0.79
**Secondary outcomes**				
Medication related secondary outcomes:				
No (%) with improvement in MAI score between baseline and 6 month follow-up	64 (39)	68 (43)	OR 1.14 (0.60 to 2.14)	0.69
No (%) with improvement in number of prescribing omissions between baseline and 6 month follow-up	28 (17)	19 (12)	OR 0.67 (0.31 to 1.46)	0.31
Mean (95% CI) MAI total score at 6 month follow-up	22 (17 to 27)	25 (20 to 29)	IRR 1.36 (0.89 to 2.08)*	0.15
Mean (95% CI) MAI total score at 12 month follow-up	25 (19 to 30)	26 (21 to 30)	IRR 1.15 (0.74 to 1.79)*	0.53
Mean (95% CI) total number of prescribing omissions at 6 month follow-up	1.1 (0.9 to 1.3)	1.1 (0.9 to 1.3)	IRR 1.06 (0.83 to 1.36)*	0.64
Mean (95% CI) total number of prescribing omissions at 12 month follow-up	1.2 (1.0 to 1.3)	0.9 (0.7 to 1.1)	IRR 0.83 (0.65 to 1.07)*	0.15
Mean (95% CI) number of medications at 6 month follow-up	7.6 (7.0 to 8.2)	7.2 (6.6 to 7.9)	MD 0.05 (−0.87 to 0.97)*	0.91
Mean (95% CI) number of medications at 12 month follow-up	8.0 (7.4 to 8.7)	7.8 (7.2 to 8.4)	MD 0.26 (−0.64 to 1.16)*	0.58
Patient reported secondary outcomes:				
Mean (95% CI) number of falls at 6 month follow-up	0.3 (0.1 to 0.4)	0.3 (0.2 to 0.4)	IRR 0.96 (0.50 to 1.84)	0.89
Mean (95% CI) number of falls at 12 month follow-up	0.2 (0.1 to 0.3)	0.2 (0.1 to 0.3)	IRR 0.90 (0.50 to 1.64)	0.74
No (%) with any fracture(s) between baseline and 6 month follow-up†	4 (3)	3 (2)	OR 0.72 (0.17 to 3.10)	0.66
No (%) with any fracture(s) between baseline and 12 month follow-up†	2 (1)	3 (2)	OR 1.51 (0.27 to 8.50)	0.64
Quality of life:				
Mean (95% CI) EQ-5D-5L utilities at 6 month follow-up‡ (inverse predicted utility)	0.2 (0.2 to 0.2)	0.2 (0.1 to 0.2)	MD −0.03 (−0.07 to 0.01)*	0.13
Mean (95% CI) EQ-5D-5L utilities at 12 month follow-up‡ (inverse predicted utility)	0.1 (0.1 to 0.2)	0.1 (0.1 to 0.2)	MD 0.00 (−0.04 to 0.03)*	0.93
Mean (95% CI) VAS at 6 month follow-up§	71 (68 to 74)	72 (69 to 74)	MD 0.53 (−2.99 to 4.06)*	0.77
Mean (95% CI) VAS at 12 month follow-up§	73 (70 to 75)	72 (70 to 75)	MD −0.42 (−3.77 to 2.93)*	0.81

AOU=Assessment of Underutilisation; CI=confidence interval; IRR=incident rate ratio; MAI=Medication Appropriateness Index; MD=mean difference; OR=odds ratio; VAS=visual analogue scale.

This table is based on multiple imputed data. Additional descriptive information on secondary outcomes by study time point can be found in supplementary table M.

* Adjusted for respective baseline score.

† Owing to low number of fractures, only binary variable (yes/no) was considered.

‡ Calculated based on German value set for EQ-5D-5L by Ludwig et al.^51^.

§ Measured by VAS of European Quality of Life-5 Dimensions questionnaire (EQ-VAS); values range from 0 to 100 with higher values indicating higher quality of life.

In the strict per protocol analysis, the adjusted odds ratio for an improvement in the MAI score between baseline and the 12 month follow-up was 1.03 (0.59 to 1.77) and the odds ratio for an improvement in the AOU was 1.25 (0.44 to 3.56) (supplementary tables H and I). The relaxed per protocol analysis produced similar results.

### Secondary outcomes

The results on whether the medication review intervention centred around the use of an eCDSS led to an improvement in the secondary outcomes, in the primary outcomes at the six month follow-up (table 4), in the per protocol analyses (supplementary table J), or in additional medication related outcomes (supplementary table K) were inconclusive. We did not find clear evidence for a difference in safety outcomes at the 12 month follow-up, but fewer safety events occurred in the intervention group at six and 12 months (supplementary table L).

## Discussion

In this cluster randomised clinical trial evaluating the effect of a structured medication review intervention supported by an electronic decision support system in older adults with multimorbidity and polypharmacy, 58.5% of patients had at least one prescribing recommendation implemented. Despite the implementation of one STOPP or START recommendation per patient on average, the results on the appropriateness of medication and the number of prescribing omissions at the 12 month follow-up compared with usual care were inconclusive, with odds ratios for the MAI and the AOU being close to one and confidence intervals being wide. However, the intervention could be safely delivered without causing harm to patients. The results on whether the medication review intervention led to an improvement in the secondary outcomes, such as the number of falls and fractures, were also inconclusive. We emphasise that given the age characteristics and the comorbidities of the study population, not all falls and fractures can be assumed to be an (adverse) effect of polypharmacy despite older adults with polypharmacy being at a higher risk for such events.^52^ ^53^ ^54^

### Strengths and limitations of study

The OPTICA trial has several strengths. Recruitment of patients was based on screening lists with random samples of each general practitioner’s patient population. The trial had minimal exclusion criteria, which resulted in the recruitment of patients who were comparable to other older patients with multimorbidity and polypharmacy in Swiss primary care.^30^ The randomisation of general practitioner clusters once the recruitment per cluster was completed helped to limit differential, selective recruitment. However, owing to the Hawthorne effect, we cannot exclude the possibility that general practitioners recruited patients with more appropriate medication regimens or more favourable unmeasurable characteristics (for example, better patient-provider relationship). A low number of patients were lost to follow-up owing to the pragmatic data collection design consisting of phone calls and electronic health record data imports. The pragmatic nature of the trial in primary care settings provides real world insights. Finally, the collection of information related to the implementation of prescribing recommendations was insightful. However, we were unable to collect this information from all general practitioners.

The trial also has several limitations. Owing to a requirement from the regulatory authority, general practitioners in the control group had to have a discussion about medication with their enrolled patients in line with usual care. General practitioners were explicitly asked not to deviate from their usual practice. Despite this, we cannot rule out the possibility that some of them diverged from their usual prescribing practice owing to the Hawthorne effect. The number of medications, however, did not differ between the two groups at any time point. The quality of the data exports from the FIRE database was challenging because of differences in how electronic health record software programs exported data. Therefore, the study team had to manually collect missing information from practices and use multiple imputation methods. As the intervention occurred at a single time point, its effectiveness may have been diluted over time. The implementation of an average of one STOPP/START recommendation per patient did not seem to affect the primary outcomes. Next, the different barriers faced by patients and providers related to the implementation of prescribing recommendations, which are numerous and well documented in the literature (for example, fears of negative health outcomes, alert fatigue),^55^ ^56^ may have led to a low implementation of recommendations. An imbalance in the MAI existed at baseline, which we attribute to chance. Therefore, we also present adjusted outcome models in the supplementary material (supplementary table E). However, all other baseline variables, including the other medication related variables, were balanced, which indicates that randomisation of the clusters worked as intended. Next, in the sample size calculation, we chose a conservative intracluster correlation coefficient to be on the safe side. Finally, STRIPA used clinical information but did not consider patients’ preferences, which may have contributed to patients’ reluctance to implement recommendations.

### Comparison with other studies

Our null findings are in line with the literature on previous clinical trials testing the effect of medication review interventions on appropriateness of medication in primary care settings. For instance, the PRIMUM trial, which randomised 72 primary care practices and 505 patients with multimorbidity and polypharmacy, did not find an improvement in the MAI after a medication review based on an eCDSS for general practitioners.^57^ This trial, however, did not assess under-prescribing and excluded patients with cognitive impairment. The results of the PRIMA-eDS trial, with 359 primary care practices and 3904 adults aged ≥75 years using multiple medications, showed that the use of an eCDSS did not lead to evidence for a between group difference in mortality or unplanned hospital admissions after a 24 month follow-up period.^25^ This study was strengthened by its longer observation period. However, it did not study medication underuse. In the OPTIMIZE trial, in which 3012 patients with dementia or mild cognitive impairment from 19 primary care clinics were randomised, the number of (potentially inappropriate) medications was similar in the two groups at the end of the six month follow-up period.^58^ These findings show that meaningful, sustainable improvements are difficult to achieve by the one-time use of an eCDSS to support medication reviews in primary care settings.

Our findings are in line with the results of previous multicentre trials evaluating an eCDSS based on the STOPP/START criteria. The SENATOR trial, in which 1536 inpatients were randomised, did not find any evidence for a between group difference in the recurrence of adverse drug events within 14 days of randomisation.^26^ Similarly, in the OPERAM trial, in which 2008 patients from 110 clusters were randomised and STRIPA was also used, the results were inconclusive as to whether the medication review intervention led to a reduction in readmission to hospital at the 12 month follow-up despite a trend towards a reduction in readmission and other clinical outcomes.^19^ The intervention came with potential cost savings of CHF3588 (£3229; €3666; $4065) per patient and a gain of 0.025 (95% confidence interval –0.002 to 0.052) quality adjusted life years per patient.^59^ Despite the more user-friendly application of the STOPP/START criteria as an eCDSS, difficulties related to implementing prescribing recommendations exist.

The low implementation rate of prescribing recommendations could, in part, account for the negative trial. However, the implementation rate was similar in previous trials. In the SENATOR trial, 15% of the overall recommendations were implemented.^26^ In the OPERAM trial, 62% of patients had at least one recommendation successfully implemented at two months.^19^ Expecting every prescribing recommendation to be implementable would be unreasonable, but the partial uptake of prescribing recommendations points to many factors that affect the overall effectiveness of medication review interventions. Our results show that the most common reasons why general practitioners reported not implementing the prescribing recommendations were that general practitioners thought that patients’ current medicines were beneficial and that recommended changes were not suitable. These findings are in line with the literature, which has shown that prescribing recommendations can be difficult to implement owing to many barriers faced by patients and prescribers.^55^ ^60^ Furthermore, the types of prescribing recommendations generated by the eCDSS were relevant for older community dwelling adults, according to two recent systematic reviews.^11^ ^61^ In the OPTICA trial, the recommendation to vaccinate patients against influenza was the most common recommendation. This was probably because influenza vaccines were not commonly recorded in patients’ medication lists.

### Implications of findings

The STRIPA web based tool was not integrated into the electronic health record software programs used by general practitioners, which could explain the challenges in implementation. The future widespread and successful use of eCDSS in primary care settings requires that limitations, such as the lack of integration of eCDSS into existing practice software and clinical workflows, must be overcome. Furthermore, in Swiss primary care settings, it will be crucial to establish an industry standard that will allow reliable exports/imports of data from electronic health record software to eCDSS, which was also one of the main challenges observed during the OPTICA trial and led to an increased expenditure of time by general practitioners who had to update the data manually in the eCDSS.

Future trials on interventions to optimise medication would benefit from interventions focusing on overcoming challenges to implementation. This not only requires preparatory qualitative studies to better understand the challenges but also use of implementation science strategies to integrate interventions into clinical workflows, tailoring interventions to users’ needs, and piloting interventions. Future interventions may benefit from being designed as repeated interventions to accommodate the dynamic prescribing practices and frequent medication changes in older patients with multimorbidity and polypharmacy.

### Conclusions

In this primary care based trial, 58.5% of patients had at least one prescribing recommendation implemented. Despite this, the results as to whether the medication review intervention centred around the use of an eCDSS led to an improvement in appropriateness of medication or a reduction in prescribing omissions at 12 months compared with a discussion about medication in line with usual care were inconclusive. Nevertheless, the intervention could be safely delivered without causing any harm to patients.

## What is already known on this topic

Inappropriate prescribing is highly prevalent in older adults with multimorbidity and polypharmacy and has been associated with adverse health outcomes
Medication review interventions might contribute to reducing inappropriate prescribing
The evidence on medication review interventions based on electronic clinical decision support systems in primary care settings is limited

## What this study adds

The structured medication review intervention based on an electronic clinical decision support system led to the implementation of certain prescribing recommendations
However, the findings as to whether the intervention led to a greater appropriateness of patients’ prescriptions overall were inconclusive

## Data Availability

The data for this study are available to other researchers on request. The data will be made available for scientific research purposes, after the proposed analysis plan has been approved. Data and documentation will be made available through a secure file exchange platform after approval of the proposal. In addition, a data transfer agreement must be signed (which defines obligations that the data requester must adhere to with regard to privacy and data handling). Deidentified participant data limited to the data used for the proposed project will be made available, along with a data dictionary and annotated case report forms. For data access, please contact the corresponding author.

## Web extra

Extra material supplied by authors

Web appendix: Supplementary materials

## Notes

Contributors: NR, SS, MSchwenkglenks, ST, MS, CS, and KTJ designed the trial. KTJ, AKA, CF, ZR, RM, NS, FV, MB, ST, MSpruit, MSchwenkglenks, NR, and SS contributed to the acquisition, analysis, and interpretation of data. KTJ and OS wrote the first draft of the manuscript. All other authors provided feedback and approved the final version of the manuscript. OS, AL, and ST provided statistical support and did the statistical analyses. NR, MSchwenkglenks, and SS obtained the funding for the OPTICA trial. NS and FV provided administrative and technical support. NR and SS supervised the conduct of the trial. KTJ and OS had full access to all the data in the study and are the guarantors. The corresponding author attests that all listed authors meet authorship criteria and that no others meeting the criteria have been omitted.

The lead authors (the manuscript’s guarantors) affirm that the manuscript is an honest, accurate, and transparent account of the study being reported; that no important aspects of the study have been omitted; and that any discrepancies from the study as originally planned (and, if relevant, registered) have been explained.

Dissemination to participants and related patient and public communities: The results of the OPTICA trial were presented at a national press conference in January 2023. The results of the OPTICA trial will be published in German and French in a journal that is commonly read by Swiss general practitioners. The submission of this article is planned for 2023. A lay summary of the OPTICA trial in German, French, and English can be found on the website of the NRP74 (https://www.nfp74.ch/de/3R0qxh8zc3RXcvXm/projekt/projekt-streit).

Provenance and peer review: Not commissioned; externally peer reviewed.
