# Supplementary material for: Optimising prescribing in older adults with multimorbidity and polypharmacy in primary care (OPTICA): cluster randomised clinical trial
Source: BMJ. 2023 May 24;381:e074054. doi: 10.1136/bmj-2022-074054 (PMC10206530; doi:10.1136/bmj-2022-074054)
Supplement: Supplementary file 1 — Web appendix: Supplementary materials [file junk074054.ww1.pdf]

Supplementary material

Optimizing Prescribing in Older Adults with Multimorbidity and Polypharmacy in Primary Care (OPTICA): A Cluster Randomized Clinical Trial (Jungo et al.)

Table of content

Appendix 1. Screenshots of the 'Systematic Tool to Reduce Inappropriate Prescribing'-Assistant (STRIPA). 2

Appendix 2. Details on the assessment of the Medication Appropriateness Index (MAI). 3

Appendix 3. Subgroup analyses. 3

Appendix 4. Additional minor deviations from the Statistical Analysis Plan (SAP). 3

Supplementary Figure A. Screening and recruitment flow chart of general practitioners and patients in the OPTICA trial, a cluster-randomized controlled trial in Swiss primary care settings. 4

Supplementary Table A. Availability of the information on the Medication Appropriateness Index (MAI) and the Assessment of Underutilization (AOU) 4

Supplementary Table B. Improvement of the primary outcomes between baseline and 12 month follow-up: Medication Appropriateness Index (MAI) and Assessment of Underutilization (AOU) 5

Supplementary Table C. Reasons for the non-implementation of prescribing recommendations, as reported by GPs in the intervention group (n=36) 5

Supplementary Table D. Descriptive primary outcomes. 5

Supplementary Figure B. Development of medication appropriateness over time: Means and 95% confidence intervals for the total Medication Appropriateness Index Score (MAI) score by study timepoint 6

Supplementary Figure C. Development of prescribing omissions over time: Means and 95% confidence intervals for the total number of prescribing omissions as measured by the Assessment of Underutilization (AOU) by study timepoint 6

Supplementary Figure D. Primary outcomes by timepoint. 7

Supplementary Table E. Adjusted primary outcome models 8

Supplementary Figure E. Subgroup analyses for the primary outcomes (n=323) 8

Supplementary Table F. Aggregated data analysis 9

Supplementary Table G. Available case analysis: Primary and secondary outcomes (n=271) 9

Supplementary Table H. Per-protocol set of participants 11

Supplementary Table I. Per-protocol analysis: Primary outcomes 11

Supplementary Table J. Per-protocol analysis: Secondary outcomes 12

Supplementary Table K. Additional secondary outcomes: Comparison between the intervention and control group 13

Supplementary Table L. Safety Outcomes: Comparison between the intervention and control group 13
Supplementary Table M. Descriptive secondary outcomes. 14

Appendix 1. Screenshots of the 'Systematic Tool to Reduce Inappropriate Prescribing'-Assistant (STRIPA).

Before using the drag/drop function:

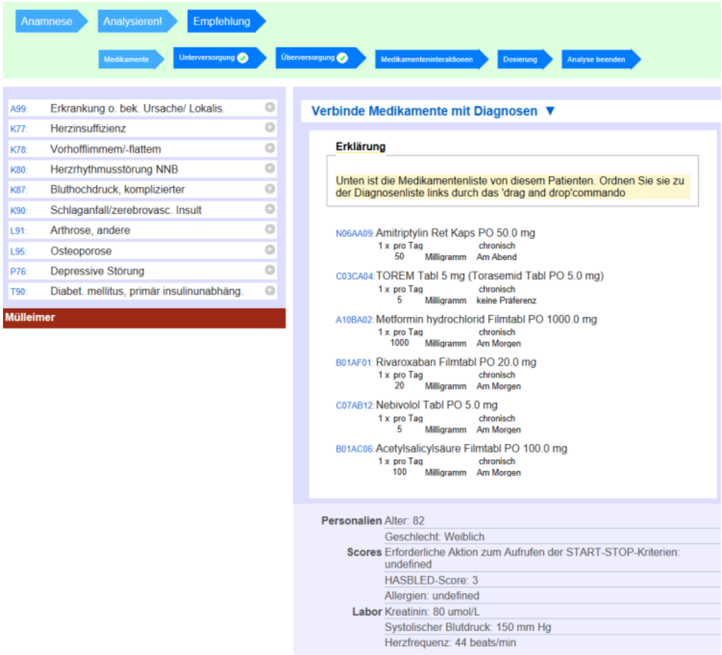

After using the drag/drop function:

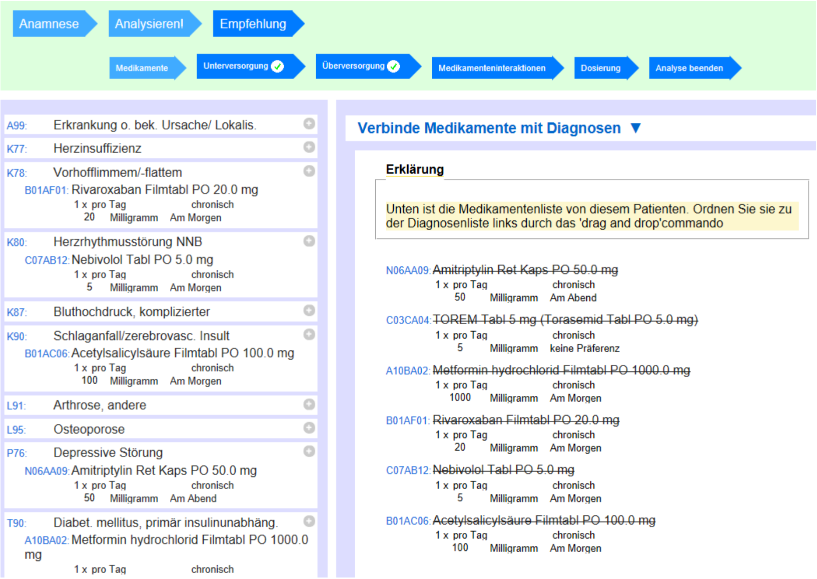

Appendix 2. Details on the assessment of the Medication Appropriateness Index (MAI).

We assessed the AOU for each non-acute condition of the patients and the MAI for each long-term medication (≥90 days, no stop date; "as needed" medications were excluded). In this trial, we used the 10-item version of the MAI, however, we excluded the cost-effectiveness item for feasibility reasons. Using data on medications, diagnoses, and lab values the assessors rated the nine remaining criteria of the MAI for each medication using a three-point scale ranging from A=appropriate, B=marginally appropriate, to C=inappropriate. Each of the nine criteria has a weight of 1-3.[1] Each "inappropriate" rating received the respective weight, while each "marginally appropriate" and "appropriate" rating was weighted with 0. This resulted in a score from 0 to 17 for each medication.

Appendix 3. Subgroup analyses.

At the GP level, the following subgroup analyses were performed: age in years (<median, ≥median), experience in years (<median, ≥median), practice size (single vs. group practice), and sex (male vs. female).

At the patient level, we performed the following subgroup analyses: number of medications (<10, ≥10), sex (male vs. female), living conditions (nursing home vs. community dwelling), age in years (65-74, 75-84, ≥85), number of chronic conditions (<median, ≥median), and willingness to deprescribe (question from rPATD 'If my doctor said it was possible, I would be willing to stop one or more of my regular medicines' – dichotomized by strongly agree/agree vs. unsure, disagree/strongly disagree).

Appendix 4. Additional minor deviations from the Statistical Analysis Plan (SAP).

We did not run the subgroup analysis related to medication adherence, since the data on medication adherence had not been collected at baseline but only at the 12 month follow-up.

The rest of the analysis is consistent with the principal features of the statistical methods described in the statistical analysis plan (SAP).

Supplementary Figure A. Screening and recruitment flow chart of general practitioners and patients in the OPTICA trial, a cluster-randomized controlled trial in Swiss primary care settings.

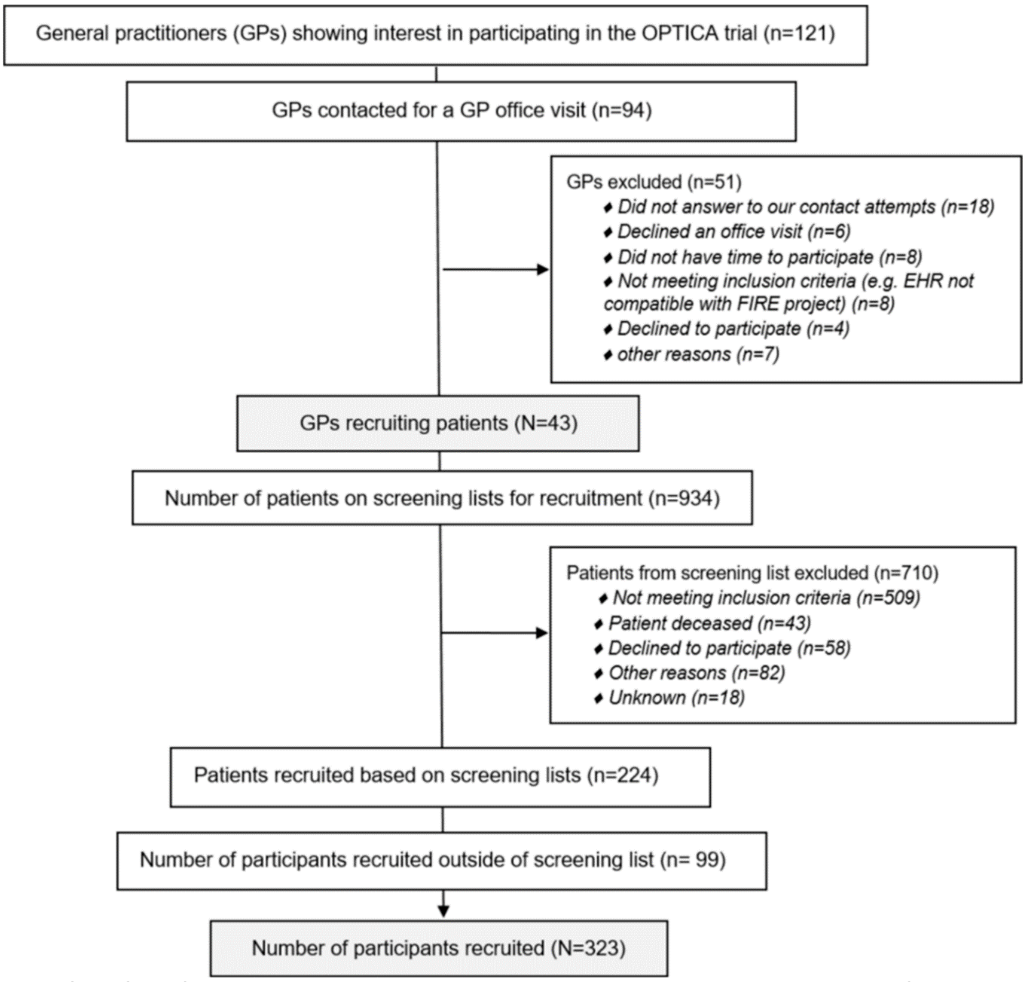

Abbreviation: OPTICA = Optimizing PharmacoTherapy in older multimorbid adults In primary CAre. | Reference: Jungo KT, Meier R, Valeri F, Schwab N, Schneider C, Reeve E, Spruit M, Schwenkglenks M, Rodondi N, Streit S. Baseline characteristics and comparability of older multimorbid patients with polypharmacy and general practitioners participating in a randomized controlled primary care trial. BMC Fam Pract. 2021 Jun 22;22(1):123. doi: 10.1186/s12875-021-01488-8. PMID: 34157981; PMCID: PMC8220761.

Supplementary Table A. Availability of the information on the Medication Appropriateness Index (MAI) and the Assessment of Underutilization (AOU)

	**All participants (n=323)**	**Control group (n=163)**	**Intervention group (n=160)**
**AOU availability**[1]			
Baseline and 12 month follow-up	265 (82%)	133 (82%)	132 (82%)
Only baseline	27 (8.4%)	14 (8.6%)	13 (8.1%)
Only 12 month follow-up	6 (1.9%)	5 (3.1%)	1 (0.6%)
Baseline and 12 month follow-up not available	25 (7.7%)	11 (6.7%)	14 (8.7%)
**MAI availability**[2]			
Baseline and 12 month follow-up	282 (87%)	143 (88%)	139 (87%)
Only baseline	19 (5.9%)	9 (5.5%)	10 (6.3%)
Only 12 month follow-up	4 (1.2%)	3 (1.8%)	1 (0.6%)

Only 6 month follow-up available	14 (4.3%)	5 (3.1%)	9 (5.6%)
Baseline and 12 month follow-up not available	4 (1.2%)	3 (1.8%)	1 (0.6%)

This table is based on the raw data. | [1]The AOU was assessed for each chronic condition at the different timepoints, as described by Jeffery et al. (Jeffery S, Ruby C, Hanlon J, et al. The impact of an interdisciplinary team on suboptimal prescribing in a long term care facility. Consult Pharm 1999;14:1386-91) | [2]The MAI was evaluated for each chronic medication at the different timepoints (excluding the cost-effectiveness item). Reference: Samsa GP, Hanlon JT, Schmader KE, et al. A summated score for the medication appropriateness index: development and assessment of clinimetric properties including content validity. J Clin Epidemiol 1994;47:891-6.

Supplementary Table B. Improvement of the primary outcomes between baseline and 12 month follow-up: Medication Appropriateness Index (MAI) and Assessment of Underutilization (AOU)

	**All participants (n=323)**	**Control group (n=163)**	**Intervention group (n=160)**
**Improvement in MAI**			
No	163 (50%)	86 (53%)	77 (48%)
Yes	119 (37%)	57 (35%)	62 (39%)
Missing	41 (13%)	20 (12%)	21 (13%)
**Improvement in AOU**			
No	227 (70%)	111 (68%)	116 (73%)
Yes	38 (12%)	22 (13%)	16 (10%)
Missing	58 (18%)	30 (18%)	28 (18%)

This table is based on the raw data.

Supplementary Table C. Reasons for the non-implementation of prescribing recommendations, as reported by GPs in the intervention group (n=36)

	N	*%*
Conviction that the current medication use is beneficial for the patient's health status	18	50
Recommendation not suitable for patient	8	22
Bad experience with previous medication changes	5	14
Changing the patient's medication does not make sense in light of his/her limited life expectancy (e.g., palliative situation)	3	8
Opinion of third parties (e.g., other prescribing physicians)	2	6
**Total**	**36**	

Legend: Despite several reminders, GPs in the intervention group only reported the reasons for why certain recommendations were not implemented for 36 recommendations, which explains the lower denominator.

Supplementary Table D. Descriptive primary outcomes.

	**Control group (n=163)**		**Intervention group (n=160)**	
	Missing n (%)	median [LQ, UQ]; mean (SD) or n (%)	Missing n (%)	median [LQ, UQ]; mean (SD) or n (%)
Medication Appropriateness Index				
MAI score total (baseline)	11 (7%)	5.0 [0.00, 38]; 27 (43)	11 (7%)	15 [4.0, 38]; 26 (28)
MAI score total (6 month follow-up)	20 (12%)	7.0 [0.00, 36]; 22 (31)	20 (13%)	14 [3.0, 38]; 25 (27)
MAI score total (12 month follow-up)	14 (9%)	8.0 [0.00, 36]; 24 (33)	19 (12%)	15 [3.0, 38]; 26 (26)
MAI improvement between baseline and 12 month follow-up	20 (12%)		21 (13%)	

no		86 (53%)		77 (48%)
yes		57 (35%)		62 (39%)
Assessment of underutilization				
Number of prescribing omissions (baseline)	16 (10%)	1.0 [0.0, 2.0]; 1.2 (1.2)	15 (9%)	1.0 [0.0, 1.0]; 0.99 (1.3)
Number of prescribing omissions (6 month follow-up)	30 (18%)	1.0 [0.0, 2.0]; 1.1 (1.2)	25 (16%)	1.0 [0.0, 2.0]; 1.0 (1.2)
Number of prescribing omissions (12 month follow-up)	25 (15%)	1.0 [0.0, 2.0]; 1.2 (1.1)	27 (17%)	1.0 [0.0, 1.0]; 0.87 (1.1)
Improvement in the AOU between baseline and 12 month follow-up	30 (18%)		28 (18%)	
no		111 (68%)		116 (73%)
yes		22 (13%)		16 (10%)

Acronyms: LQ=lower quartile, UQ=upper quartile, SD=standard deviation, MAI=Medication Appropriateness Index, AOU=Assessment of underutilization. | This table is based on the raw data.

Supplementary Figure B. Development of medication appropriateness over time: Means and 95% confidence intervals for the total Medication Appropriateness Index Score (MAI) score by study timepoint

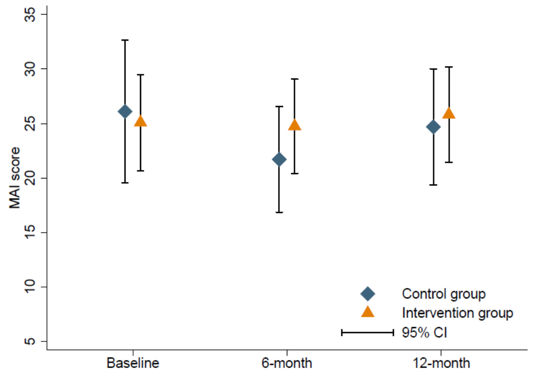

The numbers in this figure were calculated using the multiple imputed data.

Supplementary Figure C. Development of prescribing omissions over time: Means and 95% confidence intervals for the total number of prescribing omissions as measured by the Assessment of Underutilization (AOU) by study timepoint

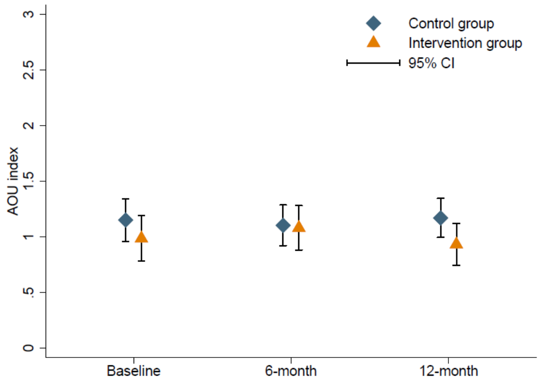

The numbers in this figure were calculated using the multiple imputed data.

Supplementary Figure D. Primary outcomes by timepoint.

Medication Appropriateness Index Score (MAI)

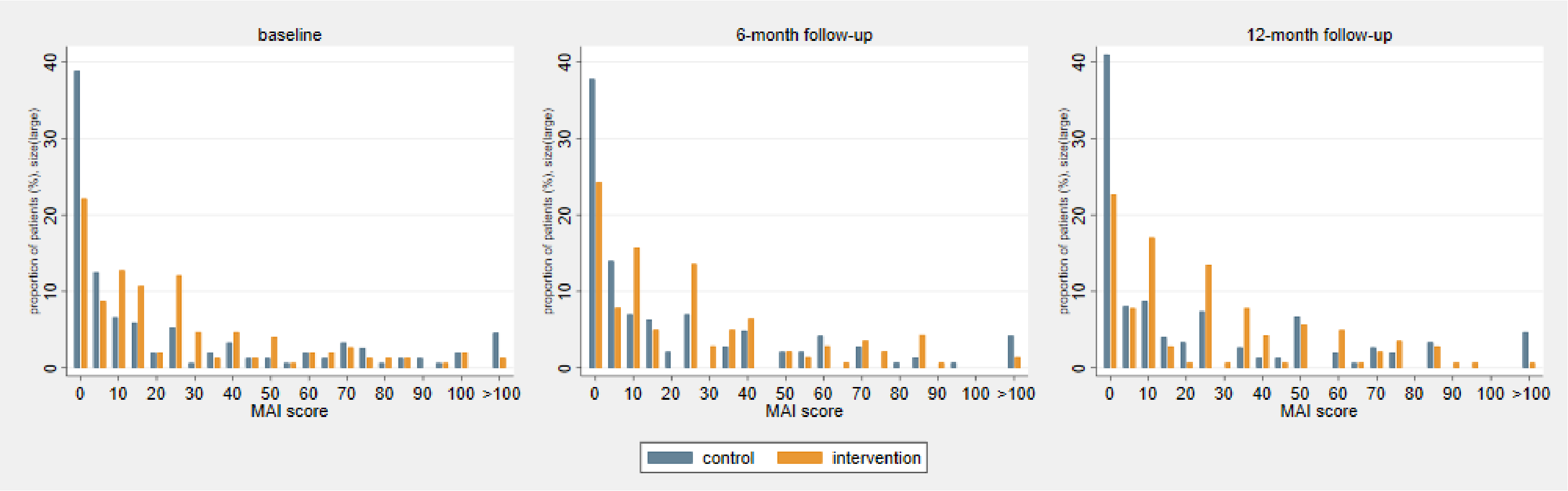

Assessment of Underutilization (AOU)

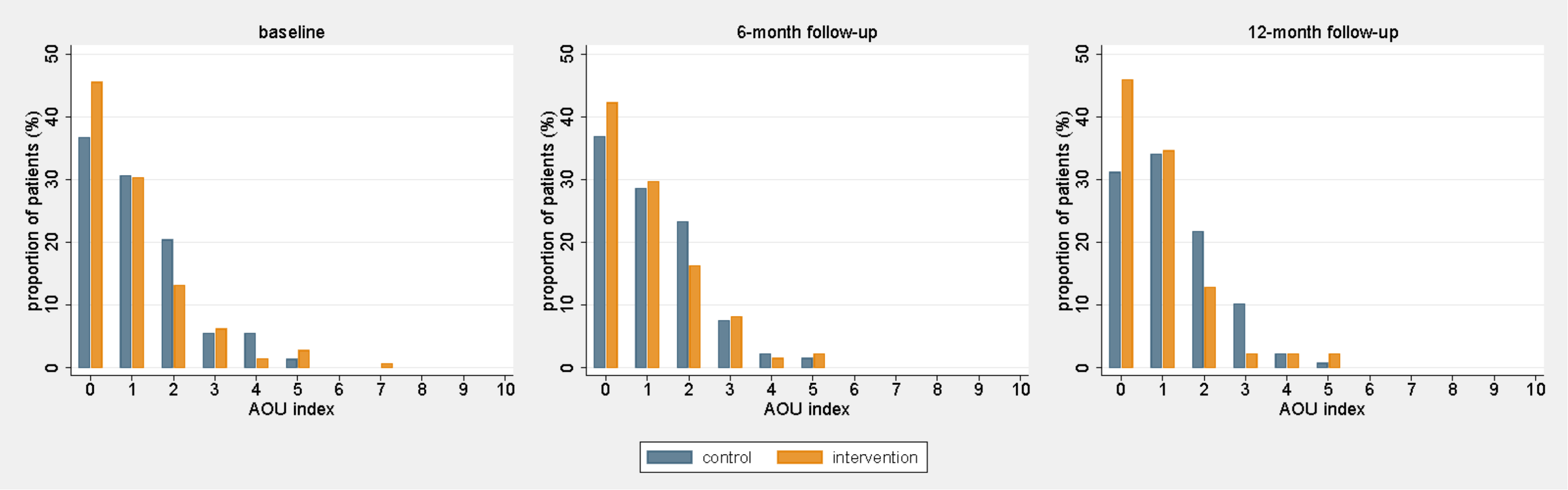

Supplementary Table E. Adjusted primary outcome models

Adjusted for baseline MAI and AOU values				
	Control (n=163)	*Intervention (n=160)*	*OR (95% CI)*	*p value*
	n (%)			
Improvement in the MAI score between baseline and 12 month follow-up	57 (40%)	62 (45%)	1.01 (0.58 to 1.75)	0.97
Improvement in the AOU between baseline and 12 month follow-up	22 (17%)	16 (12%)	1.07 (0.43 to 2.64)	0.88
Adjusted for all subgroup variables[1]				
	n (%)		*OR (95% CI)*	
Improvement in the MAI score between baseline and 12 month follow-up	67 (41%)	68 (43%)	1.36 (0.73 to 2.53)	0.33
Improvement in the AOU between baseline and 12 month follow-up	28 (17%)	24 (15%)	0.91 (0.35 to 2.39)	0.85

AOU=Assessment of Underutilization, CI=Confidence Interval, MAI=Medication Appropriateness Index, OR=Odds Ratio. | Calculated using multiple imputed data. | [1]Models were adjusted for number of chronic medications, patient's gender, permanent nursing home stay (yes/no), number of chronic conditions, patients' willingness to have medications deprescribed, patients' age, practice form, GPs' gender, GPs' age, and GPs' experience. All these variables were assessed at baseline.

Supplementary Figure E. Subgroup analyses for the primary outcomes (n=323)

Part A) Improvement in medication appropriateness between baseline and 12 month follow-up as measured by the Medication Appropriateness Index (MAI)

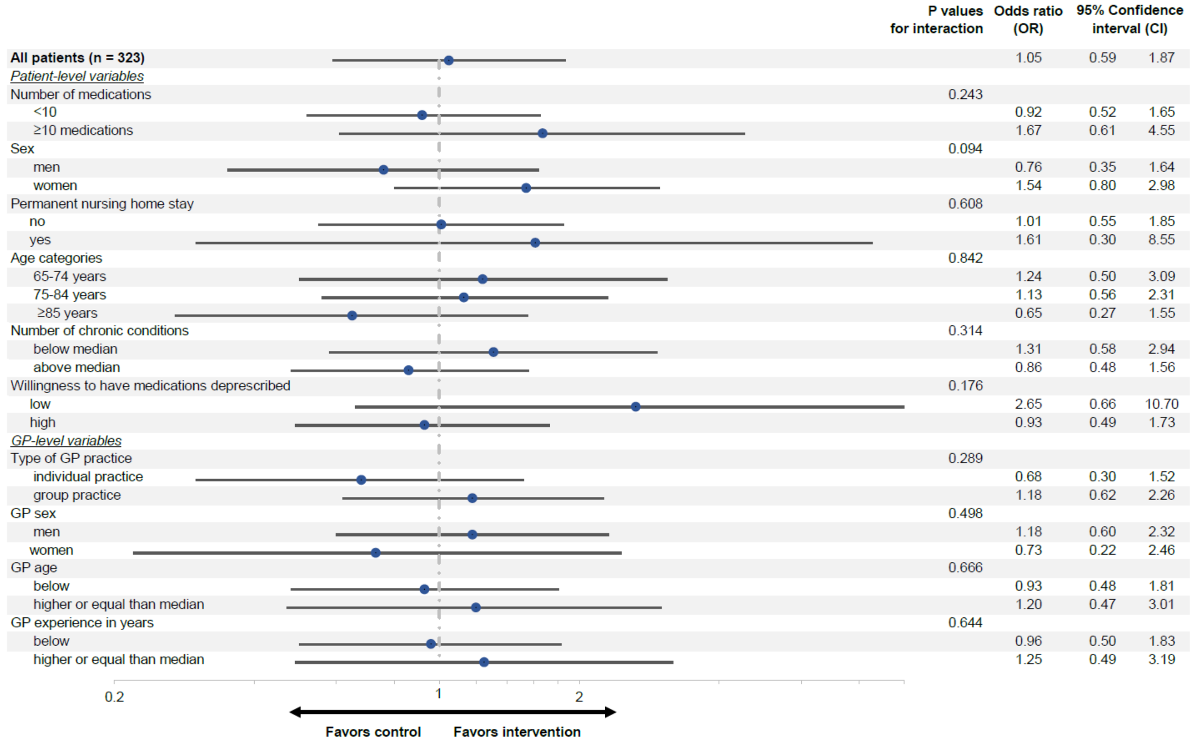

Part B) Improvement in prescribing omissions between baseline and 12 month follow-up as measured by the Assessment of Underutilization

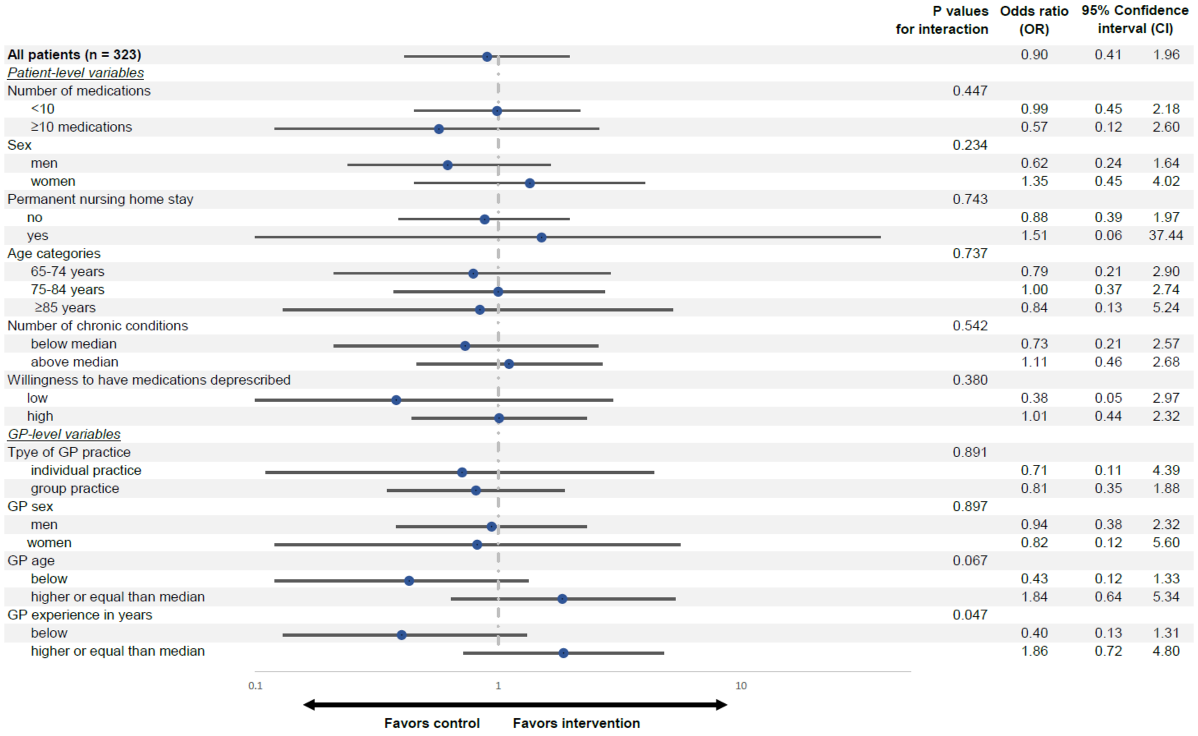

Sub-group analyses as specified in the Statistical Analysis Plan. | Forest plots are displayed on a logarithmic scale.

Supplementary Table F. Aggregated data analysis

	Control (n=22 GPs)	*Intervention (n=21 GPs)*	*Percentage mean difference (95% CI)*	*P value*
	Mean percentage (95% CI)			
Improvement in the MAI score between baseline and 12 month follow-up	41.0 (26.9 to 55.2)	41.6 (30.8 to 52.5)	0.61 (-16.76 to 17.98)	0.94
Improvement in the AOU between baseline and 12 month follow-up	19.0 (7.2 to 30.8)	13.4 (6.1 to 20.7)	-5.64 (-19.45 to 8.18)	0.42

AOU=Assessment of Underutilization, CI=Confidence Interval, MAI=Medication Appropriateness Index, OR=Odds Ratio. | Calculated using multiple imputed data.

Supplementary Table G. Available case analysis: Primary and secondary outcomes (n=271)

	Control	*Intervention*		*P value*
**Primary outcomes**	*n (%)*		*OR (95% CI)*	
Improvement in the MAI score between baseline and 12 month follow-up	57/143 (40%)	62/139 (45%)	1.19 (0.67 to 2.12)	0.55
Improvement in the AOU between baseline and 12 month follow-up	22/133 (17%)	16/132 (12%)	0.72 (0.32 to 1.63)	0.43
**Secondary outcomes**				

Medication-related secondary outcomes				
	n (%)		*OR (95% CI)*	
Improvement in the MAI score between baseline and 6 month follow-up	53/140 (38%)	62/139 (45%)	1.34 (0.69 to 2.63)	0.389
Improvement in the AOU between baseline and 6 month follow-up	19/128 (15%)	15/134 (11%)	0.74 (0.32 to 1.70)	0.479
	Mean (95% CI)		*IRR (95% CI)* [4]	
MAI total score at 6 month follow-up	21.8 (16.7; 27.0)	25.3 (20.9; 29.8)	1.33 (0.85 to 2.07)	0.216
MAI total score at 12 month follow-up	23.6 (18.2; 29.0)	26.3 (21.9; 30.7)	1.11 (0.68 to 1.81)	0.682
Total number of prescribing omissions at 6 month follow-up as measured by the AOU	1.1 (0.9; 1.3)	1.0 (0.8; 1.2)	1.02 (0.78 to 1.33)	0.878
Total number of prescribing omissions at 12 month follow-up as measured by the AOU	1.2 (1.0; 1.4)	0.9 (0.7; 1.1)	0.82 (0.64 to 1.06)	0.136
	Mean (95% CI)		*MD (95% CI)* [4]	
Number of medications at 6 month follow-up	8 (7; 8)	7 (7; 8)	0.32 (-0.33 to 0.96)	0.334
Number of medications at 12 month follow-up	8 (7; 8)	8 (7; 9)	0.43 (-0.38 to 1.23)	0.300
Patient-reported secondary outcomes				
	Mean (95% CI)		*IRR (95% CI)* [4]	
Number of falls at 6 month follow-up	0.3 (0.1; 0.4)	0.3 (0.2; 0.4)	0.92 (0.46 to 1.83)	0.816
Number of falls at 12 month follow-up	0.2 (0.1; 0.3)	0.2 (0.1; 0.3)	0.84 (0.47 to 1.51)	0.566
	n (%)		*OR (95% CI)*	
Any fracture(s) between baseline and 6 month follow-up[1]	4 (3%)	3 (2%)	0.72 (0.17 to 3.10)	0.660
Any fracture(s) between baseline and 12 month follow-up[1]	2 (1%)	3 (2%)	1.51 (0.27 to 8.50)	0.643
Quality of life				
	Mean (95% CI)		*MD (95% CI)* [4]	
EQ-5D-5L utilities at 6 month follow-up[2] (inverse predicted utility – IPU)	0 (0 to 0)	0 (0 to 0)	-0.03 (-0.07 to 0.01)	0.139
EQ-5D-5L utilities at 12 month follow-up[2] (inverse predicted utility – IPU)	0 (0 to 0)	0 (0 to 0)	0.00 (-0.03 to 0.04)	0.915
Visual analogue scale at 6 month follow-up[3]	71 (68; 74)	72 (69; 75)	0.88 (-2.43 to 4.18)	0.603
Visual analogue scale at 12 month follow-up[3]	73 (70; 75)	73 (71; 76)	-0.09 (-3.28 to 3.09)	0.955

This table is based on the raw data. | Acronyms: AOU=Assessment of Underutilization, CI=Confidence Interval, IRR=Incident Rate Ratio, MAI=Medication Appropriateness Index, MD=Mean Difference, OR=Odds Ratio. This table is based on multiple imputed data. | [1]Due to the low number of fractures only the binary variable (yes/no) was considered. | [2]Calculated using the German Value Set for the EQ-5D-5L by Ludwig et al. (Ludwig, K., Graf von der Schulenburg, JM. & Greiner, W. German Value Set for the EQ-5D-5L. PharmacoEconomics 36, 663–674 (2018). https://doi.org/10.1007/s40273-018-0615-8) | [3]Measured by visual analogue scale of the European Quality of Life-5 Dimensions questionnaire (EQ-VAS). Values range from 0 to 100 with higher values indicating a higher quality of

life. | [4]Adjusted for baseline characteristics.

Supplementary Table H. Per-protocol set of participants

Strict per-protocol set of patients. [1]			
	Randomized (n=323)	*Intervention (n=160)*	*Control (n=163)*
Not receiving allocated intervention	26	13	13
Cluster <4	5	0	5
Age <65 years	0	0	0
Number of recorded chronic conditions in the electronic health records <3 [2]	50	27	23
Number of recorded medications in the electronic health records <5 [2]	83	47	36
PPset total (n=194)	194	97	97

Relaxed per-protocol set of patients. [1]			
	Randomized (n=323)	*Intervention (n=160)*	*Control (n=163)*
Not receiving intervention	26	13	13
Cluster <4	5	0	5
Age <65 years	0	0	0
Number of recorded chronic conditions in the electronic health records <1 [2]	31	15	16
Number of recorded medications in the electronic health records <1 [2]	22	11	11
PPset total (n=261)	261	138	123

[1] Multiple criteria can apply. | [2] During the study period.

Supplementary Table I. Per-protocol analysis: Primary outcomes

	Control	*Intervention*	*OR (95% CI)*	*P value*
**Strict per-protocol analysis** *(n=194, clusters=39)*	*N=97*	*N=97*		
Improvement in the MAI score between baseline and 12 month follow-up	44 (46%)	45 (47%)	1.03 (0.59 to 1.77)	0.93
Improvement in the AOU between baseline and 12 month follow-up	10 (10%)	12 (12%)	1.25 (0.44 to 3.56)	0.68
**Relaxed per-protocol analysis** *(n=261, clusters=43)*	*N=123*	*N=138*		
Improvement in the MAI score between baseline and 12 month follow-up	47 (39%)	58 (42%)	1.18 (0.67 to 2.07)	0.57
Improvement in the AOU between baseline and 12 month follow-up	17 (14%)	20 (14%)	1.09 (0.48 to 2.47)	0.84

Acronyms: AOU=Assessment of Underutilization, CI=Confidence Interval, MAI=Medication Appropriateness Index, OR=Odds Ratio. | Calculated using multiple imputed data.

Supplementary Table J. Per-protocol analysis: Secondary outcomes

	Control (n=163)	*Intervention (n=160)*		*P value*
**Medication-related secondary outcomes**				
	n (%)		*OR (95% CI)*	
Improvement in the MAI score between baseline and 6 month follow-up	42 (44%)	47 (48%)	1.23 (0.62 to 2.44)	0.56
Improvement in the AOU between baseline and 6 month follow-up	14 (14%)	11 (11%)	0.80 (0.29 to 2.17)	0.66
	Mean (95% CI)		*IRR (95% CI)* [4]	
MAI total score at 6 month follow-up	23 (17; 29)	27 (22; 33)	1.87 (1.19 to 2.95)	0.007
MAI total score at 12 month follow-up	24 (18; 31)	29 (23; 34)	1.50 (0.94 to 2.38)	0.09
Total number of prescribing omissions at 6 month follow-up as measured by the AOU	1 (1; 1)	1 (1; 1)	1.07 (0.77 to 1.48)	0.69
Total number of prescribing omissions at 12 month follow-up as measured by the AOU	1 (1; 1)	1 (1; 1)	0.82 (0.60 to 1.12)	0.22
	Mean (95% CI)		*Mean difference (95% CI)* [4]	
Number of medications at 6 month follow-up	8 (7; 9)	8 (8; 9)	0.51 (-0.30 to 1.32)	0.22
Number of medications at 12 month follow-up	8 (8; 9)	9 (8; 10)	0.55 (-0.51 to 1.61)	0.31
**Patient-reported secondary outcomes**				
	Mean (95% CI)		*IRR (95% CI)* [4]	
Number of falls at 6 month follow-up	0 (0; 0)	0 (0; 0)	0.67 (0.28 to 1.60)	0.36
Number of falls at 12 month follow-up	0 (0; 0)	0 (0; 0)	0.95 (0.45 to 1.98)	0.89
	Mean (95% CI)		*OR (95% CI)*	
Any fracture(s) between baseline and 6 month follow-up[3]	4 (5%)	1 (1%)	0.22 (0.02 to 1.91)	0.17
Any fracture(s) between baseline and 12 month follow-up [3]	2 (2%)	2 (2%)	0.89 (0.13 to 6.11)	0.90
Quality of life				
	Mean (95% CI)		*Mean difference (95% CI)* [4]	
EQ-5D-5L utilities at 6 month follow-up[1] (inverse predicted utility – IPU)	0 (0 to 0)	0 (0 to 0)	-0.05 (-0.11 to 0.01)	0.07
EQ-5D-5L utilities at 12 month follow-up[1] (inverse predicted utility – IPU)	0 (0 to 0)	0 (0 to 0)	0.00 (-0.05 to 0.04)	0.90
Visual analogue scale at 6	70 (67; 74)	72 (69; 76)	2.67 (-1.79 to 7.14)	0.24

month follow-up[2]				
Visual analogue scale at 12 month follow-up[2]	72 (69; 75)	73 (70; 76)	1.82 (-2.21 to 5.84)	0.38

Acronyms: AOU=Assessment of Underutilization, CI=Confidence Interval, IRR=Incident Rate Ratio, MAI=Medication Appropriateness Index, MD=Mean difference, OR=Odds Ratio. This table is based on multiple imputed data and the strict per-protocol set of patients. | [1]Calculated based on the German Value Set for the EQ-5D-5L by Ludwig et al. (Ludwig, K., Graf von der Schulenburg, JM. & Greiner, W. German Value Set for the EQ-5D-5L. PharmacoEconomics 36, 663–674 (2018). https://doi.org/10.1007/s40273-018-0615-8) | [2]Measured by visual analogue scale of the European Quality of Life-5 Dimensions questionnaire (EQ-VAS). Values range from 0 to 100 with higher values indicating a higher quality of life. | [3]Due to the low number of fractures a binary variable (any fracture yes/no) was considered. | [4]Adjusted for baseline characteristics.

Supplementary Table K. Additional secondary outcomes: Comparison between the intervention and control group

	Control (n=163)	*Intervention (n=160)*		*P value*
**Additional secondary outcomes**				
	Number of events (%)[3]		*IRR (95% CI)[4]*	
Averaged MAI at 6 month follow-up[1]	140 (20)	143 (20)	1.42 (0.95 to 2.12)	0.08
Averaged MAI at 12 month follow-up[1]	141 (19)	149 (14)	1.29 (0.84 to 1.98)	0.25
Averaged number of prescribing omissions at the 6 month follow-up[2]	135 (25)	133 (30)	0.92 (0.65 to 1.29)	0.63
Averaged number of prescribing omissions at the 12 month follow-up[2]	133 (27)	138 (25)	0.74 (0.53 to 1.04)	0.08

Acronyms: AOU=Assessment of Underutilization, CI=Confidence Interval, IRR=Incident Rate Ratio, MAI=Medication Appropriateness Index. This table is based on multiple imputed data. | [1]The total MAI score divided by the total number of chronic medications. | [2]The total number of prescribing omissions divided by the total number of chronic conditions. | [3]Number of patients with data (with multiple imputations). | [4]Adjusted for baseline characteristics.

Supplementary Table L. Safety Outcomes: Comparison between the intervention and control group

	Control (n=163)	*Intervention (n=160)*	*P value*
**Additional secondary outcomes**			
	Number of events (%)		
Any emergency room visits at baseline			0.63
No	127 (78%)	131 (82%)	
Yes	30 (18%)	26 (16%)	
Missing	6 (4%)	3 (2%)	
Any emergency room visits at the 6 month follow-up			0.05
No	120 (74%)	127 (79%)	
Yes	26 (16%)	15 (9.4%)	
Missing	17 (10%)	18 (11%)	
Any emergency room visits at the 12 month follow-up			0.44
No	127 (78%)	126 (79%)	
Yes	19 (12%)	15 (9.4%)	
Missing	17 (10%)	19 (12%)	
Unplanned hospitalizations at baseline			0.44
No	130 (80%)	138 (86%)	

Yes	25 (15%)	20 (13%)	
Missing	7 (4%)	1 (1%)	
Unplanned hospitalizations at the 6 month follow-up			0.02
No	127 (78%)	137 (86%)	
Yes	18 (11%)	6 (3.8%)	
Missing	18 (11%)	17 (11%)	
Unplanned hospitalizations at the 12 month follow-up			0.88
No	131 (80%)	128 (80%)	
Yes	15 (9.2%)	14 (8.7%)	
Missing	17 (10%)	18 (11%)	
Death during the 12 month follow-up period			0.97
No	157 (96%)	154 (96%)	
Yes	6 (3.7%)	6 (3.8%)	

The P values were computed from an unadjusted robust generalized estimating equation (GEE) model.

Supplementary Table M. Descriptive secondary outcomes.

	**Control group (n=163)**		**Intervention group (n=160)**	
	Missing n (%)	*median [IQR]; mean (sd) or n (%)*	*Missing n (%)*	*median [IQR]; mean (sd) or n (%)*
Falls				
Number of falls in the last 6 months (baseline)	10 (6%)		5 (3%)	
0		128 (79%)		125 (78%)
1		19 (12%)		25 (16%)
2		4 (2.5%)		3 (1.9%)
>2		2 (1.2%)		2 (1.3%)
Number of falls in the last 6 months (6 month follow-up)	17 (10%)		18 (11%)	
0		121 (74%)		114 (71%)
1		20 (12%)		19 (12%)
2		2 (1.2%)		8 (5%)
>2		3 (1.8%)		1 (0.6%)
Number of falls in the last 6 months (12 month follow-up)	20 (12%)		20 (13%)	
0		118 (72%)		119 (74%)
1		21 (13%)		17 (11%)
2		3 (1.8%)		2 (1.3%)
>2		1 (0.6%)		2 (1.3%)
Fractures				
Number of fractures in the last 6 months (baseline)	8 (5%)		4 (3%)	
0		152 (93%)		152 (95%)
1		3 (1.8%)		4 (2.5%)
2		0 (0%)		0 (0%)
>2		0 (0%)		0 (0%)
Number of fractures in the last 6 months (6 month follow-up)	18 (11%)		18 (11%)	
0		141 (87%)		139 (87%)
1		2 (1.2%)		3 (1.9%)
2		0 (0%)		0 (0%)

>2		2 (1.2%)		0 (0%)
Number of fractures in the last 6 months (12 month follow-up)	20 (12%)		19 (12%)	
0		141 (87%)		138 (86%)
1		2 (1.2%)		3 (1.9%)
2		0 (0%)		0 (0%)
>2		0 (0%)		0 (0%)
Quality of life[1]				
EQ-5D-5L – Mobility (baseline)	16 (10%)		8 (5%)	
No problems		59 (36%)		69 (43%)
Slight problems		35 (21%)		34 (21%)
Moderate problems		32 (20%)		28 (17%)
Severe problem		20 (12%)		21 (13%)
Unable		1 (0.61%)		0 (0.00%)
EQ-5D-5L – Mobility (6 month follow-up)	26 (16%)		22 (14%)	
No problems		50 (31%)		58 (36%)
Slight problems		36 (22%)		37 (23%)
Moderate problems		35 (21%)		33 (21%)
Severe problem		15 (9.2%)		8 (5.0%)
Unable		1 (0.61%)		2 (1.3%)
EQ-5D-5L – Mobility (12 month follow-up)	25 (15%)		25 (16%)	
No problems		63 (39%)		75 (47%)
Slight problems		37 (23%)		37 (23%)
Moderate problems		32 (20%)		18 (11%)
Severe problem		5 (3.1%)		5 (3.1%)
Unable		1 (0.61%)		0 (0.00%)
EQ-5D-5L – Selfcare (baseline)	16 (10%)		8 (5%)	
No problems		124 (76%)		134 (84%)
Slight problems		13 (8.0%)		11 (6.9%)
Moderate problems		7 (4.3%)		4 (2.5%)
Severe problem		2 (1.2%)		2 (1.3%)
Unable		1 (0.61%)		1 (0.63%)
EQ-5D-5L – Selfcare (6 month follow-up)	26 (16%)		22 (14%)	
No problems		119 (73%)		118 (74%)
Slight problems		10 (6.1%)		15 (9.4%)
Moderate problems		6 (3.7%)		3 (1.9%)
Severe problem		1 (0.61%)		1 (0.63%)
Unable		1 (0.61%)		1 (0.63%)
EQ-5D-5L – Selfcare (12 month follow-up)	25 (15%)		25 (16%)	
No problems		120 (74%)		116 (73%)
Slight problems		13 (8.0%)		13 (8.1%)
Moderate problems		3 (1.8%)		6 (3.8%)
Severe problem		2 (1.2%)		0 (0.00%)
Unable		0 (0.00%)		0 (0.00%)
EQ-5D-5L – Activities (baseline)	16 (10%)		8 (5%)	
No problems		99 (61%)		104 (65%)
Slight problems		19 (12%)		16 (10%)

Moderate problems		24 (15%)		22 (14%)
Severe problem		3 (1.8%)		8 (5.0%)
Unable		2 (1.2%)		2 (1.3%)
EQ-5D-5L – Activities (6 month follow-up)	26 (16%)		22 (14%)	
No problems		75 (46%)		82 (51%)
Slight problems		34 (21%)		31 (19%)
Moderate problems		18 (11%)		20 (13%)
Severe problem		8 (4.9%)		4 (2.5%)
Unable		2 (1.2%)		1 (0.63%)
EQ-5D-5L – Activities (12 month follow-up)	25 (15%)		26 (16%)	
No problems		90 (55%)		96 (60%)
Slight problems		25 (15%)		25 (16%)
Moderate problems		18 (11%)		11 (6.9%)
Severe problem		5 (3.1%)		2 (1.3%)
Unable		0 (0.00%)		0 (0.00%)
EQ-5D-5L – Pain or discomfort (baseline)	16 (10%)		8 (5%)	
No problems		61 (37%)		60 (38%)
Slight problems		31 (19%)		33 (21%)
Moderate problems		41 (25%)		43 (27%)
Severe problem		14 (8.6%)		13 (8.1%)
Unable		0 (0.00%)		3 (1.9%)
EQ-5D-5L – Pain or discomfort (6 month follow-up)	26 (16%)		22 (14%)	
No problems		25 (15%)		30 (19%)
Slight problems		51 (31%)		47 (29%)
Moderate problems		43 (26%)		50 (31%)
Severe problem		17 (10%)		11 (6.9%)
Unable		1 (0.61%)		0 (0.00%)
EQ-5D-5L – Pain or discomfort (12 month follow-up)	25 (15%)		25 (16%)	
No problems		40 (25%)		41 (26%)
Slight problems		53 (33%)		46 (29%)
Moderate problems		37 (23%)		37 (23%)
Severe problem		8 (4.9%)		11 (6.9%)
Unable		0 (0.00%)		0 (0.00%)
EQ-5D-5L – Anxiety (baseline)	17 (10%)		8 (5%)	
No problems		95 (58%)		99 (62%)
Slight problems		29 (18%)		33 (21%)
Moderate problems		19 (12%)		18 (11%)
Severe problem		3 (1.8%)		2 (1.3%)
Unable		0 (0.00%)		0 (0.00%)
EQ-5D-5L – Anxiety (6 month follow-up)	26 (16%)		22 (14%)	
No problems		77 (47%)		84 (52%)
Slight problems		40 (25%)		29 (18%)
Moderate problems		15 (9.2%)		22 (14%)
Severe problem		4 (2.5%)		3 (1.9%)
Unable		1 (0.61%)		0 (0.00%)
EQ-5D-5L – Anxiety (12 month follow-up)	25 (15%)		25 (16%)	
No problems		95 (58%)		88 (55%)
Slight problems		30 (18%)		34 (21%)

Moderate problems		12 (7.4%)		12 (7.5%)
Severe problem		1 (0.61%)		1 (0.63%)
Unable		0 (0.00%)		0 (0.00%)
EQ-5D-5L utilities[2]				
Baseline	17 (10%)	0.89 [0.81, 0.94]; 0.83 (0.18)	8 (5%)	0.89 [0.77, 0.97]; 0.83 (0.20)
6 month follow-up	26 (16%)	0.86 [0.77, 0.92]; 0.79 (0.22)	22 (14%)	0.87 [0.80, 0.94]; 0.83 (0.18)
12 month follow-up	25 (15%)	0.91 [0.82, 0.94]; 0.86 (0.14)	26 (16%)	0.91 [0.83, 0.94]; 0.87 (0.15)
EQ-5D-5L - Visual analogue scale (VAS)[3]				
Baseline VAS	17 (10%)	70 [60, 80]; 70 (17)	10 (6%)	75 [60, 80]; 71 (17)
6 month follow-up VAS	26 (16%)	75 [60, 85]; 71 (17)	22 (14%)	75 [60, 80]; 72 (16)
12 month follow-up VAS	25 (15%)	75 [60, 80]; 73 (15)	27 (17%)	75 [60, 85]; 73 (15)

Acronyms: IQR=inter quartile range, SD=standard deviation, MAI=Medication Appropriateness Index, AOU=Assessment of underutilization, VAS=Visual Analogue Scale. | This table is based on the raw data. | [1]Quality of Life was assessed using the EQ-5D-5L questionnaire of the EuroQol group. | [2]Calculated based on the German Value Set for the EQ-5D-5L by Ludwig et al. (Ludwig, K., Graf von der Schulenburg, JM. & Greiner, W. German Value Set for the EQ-5D-5L. PharmacoEconomics 36, 663–674 (2018). https://doi.org/10.1007/s40273-018-0615-8) | [3]Measured by visual analogue scale of the European Quality of Life-5 Dimensions questionnaire (EQ-VAS). Values range from 0 to 100 with higher values indicating a higher quality of life.

Reference:

1. Samsa GP, Hanlon JT, Schmader KE, et al. A summated score for the medication appropriateness index: development and assessment of clinimetric properties including content validity. *Journal of clinical epidemiology*. Aug 1994;47(8):891-6.
